# Qualitative and quantitative study of the highly specialized lipid tissues of cetaceans using HR-MAS NMR and classical GC

**DOI:** 10.1371/journal.pone.0180597

**Published:** 2017-07-05

**Authors:** Jean-Luc Jung, Gaelle Simon, Eric Alfonsi, Didier Thoraval, Nelly Kervarec, Douraied Ben Salem, Sami Hassani, Frédéric Domergue

**Affiliations:** 1 BioGeMME, UFR Sciences et Techniques, Université de Brest, Brest, France; 2 Plateforme Technologique de Résonance Magnétique Nucléaire, Résonance Paramagnétique Électronique et Spectrométrie de Masse, Université de Brest, Brest, France; 3 Océanopolis, Port de Plaisance du Moulin Blanc, Brest, France; 4 Laboratoire de Biogenèse Membranaire, UMR 5200, CNRS, Université de Bordeaux, Villenave d’Ornon, France; 5 Unit of Forensic Imaging, LaTIM, UMR 1101, INSERM, University Hospital of Brest, Boulevard Tanguy Prigent, Brest, France; Institute of Deep-sea Science and Engineering, Chinese Academy of Sciences, CHINA

## Abstract

Cetacean adipose tissues contain an extremely very wide variety of acyl-chains present in triacylglycerols and / or wax esters. In addition, changes in the lipid composition across organs suggest fine stratification. It therefore remains technically challenging to describe precisely the lipid organization of these tissues. In the present study, we used in parallel HR-MAS NMR (High Resolution Magic Angle Spinning Nuclear Magnetic Resonance) and GC (gas-chromatography) to characterize and quantify the lipids and fatty acyl-chains from the blubber and melon of two odontocete species. Both methods generated very similar compositions, but each presented clear advantages. While GC underestimated the amount of short branched fatty acyl-chains, which are specific to cetacean adipose tissues and most probably of primary importance for their functioning, HR-MAS NMR allowed for their exact quantification. Conversely, when HR-MAS NMR could only discriminate a few types of fatty acyl-chain families, GC unambiguously identified and quantified most of them. In addition, this technique allowed for the determination of the wax esters molecular species. Our results further suggest that the stratification of these adipose tissues relies on changes in the triacylglycerol to wax ester ratio and in the fatty acyl composition of triacylglycerols, but not on changes in the wax esters composition. Altogether, our data show that the complementarities of these two approaches result in lipid analyses of unprecedented precision, paving the way for the detailed description of the fatty acyl composition of cetacean adipose tissues and the understanding of their functioning.

## Introduction

Adipose tissues having highly specialized functions are one of the major adaptations of cetaceans to the marine environment. The blubber is a particular hypodermis which can represent a significant part of the body mass. It has multifunctional roles like metabolic energy storage, insulation and structuring of the hydrodynamic shape of the body [1,2]. The blubber has a polyphyletic origin and belongs to cetaceans as well as to pinnipeds and sirenians. The melon is another lipid-rich tissue, only found in the head of certain cetaceans, i.e. the odontocetes. This adipose tissue allows them to emit, organize and focus in the environment high-frequency sounds produced by nasal structures called MLDB (monkey lips dorsal bursae) complex [3–5]. The melon together with mandibular fats, another adipose tissue implied in the reception of the sound, form the so-called acoustic tissues that are important for echolocation [6]. These adipose tissues (blubber and acoustic tissues) show very specific lipid compositions, which must be of first importance for their functioning.

In mammals, very common saturated and monounsaturated FA (fatty acids) with 16 and 18 carbon atoms, together with various proportions of dietary FA, are usually accumulated in adipose tissues [7]. In contrast, the melons and blubbers of most cetaceans are rich in specific FA, generally not found in mammals, like isobranched FA (*i*-FA) [5,6,8–14]. In particular, IVA (isovaleric acid), a short-chain *i*-FA (*i*-5:0), is commonly detected in the adipose tissues of odontocetes (but neither in mysticets nor in other marine mammals, like pinnipeds and sirenians) [15]. IVA is synthesized from an intermediate of the leucine catabolism [16], and its accumulation is highly toxic to other mammals [17]. However, odontocetes, especially delphinids, monodontids and phocoenids, can accumulate high concentrations of IVA, in both blubber and acoustic tissues. In some species, IVA represents up to half of the blubber and melon FA [11,18]. The presence of IVA in acoustic tissues appears to be concomitant with the presence of isopentadecanoic acid (*i*-15:0). Other odontocetes, like beaked whales in which IVA is only present at low concentrations, accumulate instead medium-chain *i*-FA, like isolauric acid (*i*-12:0) in their acoustic tissues [19,20]. Finally, other short-chain FA like IBA (isobutyric acid, *iso*-4:0) or 2-MBA (2-methyl-butyric acid, *anteiso*-5:0) have also been detected, but more rarely, as for example in melons from the harbour porpoise or the stripped dolphin [8,10,11,13].

Cetaceans synthesize and accumulate these unusual FA in TAG (triacylglycerols) and/or in WE (wax esters), these neutral lipids generally representing > 95% of all lipids founds of adipose tissues [2,12,14,21]. TAG consist of a glycerol esterified to three fatty acids, while WE are made of a fatty alcohol esterified to a fatty acid. The proportion of these two neutral lipid classes is depending on the species, the age of the animals, the organ considered as well as on the diet, indicating that, if TAG and WE metabolisms are more or less generalized among odontocetes, they are regulated in complex ways [14,22]. A further degree of complexity is reached when considering that blubbers and melons are highly organized and stratified tissues so that the FA composition varies drastically within each organ. For example, in most odontocetes species, the inner part of the blubber, close to muscle of the animal, is usually richer in longer, straight-chain FA, while higher concentrations of IVA are found in the outer blubber [10,19,23]. McLellan *et al*. [24] hypothesized that the blubber of small cetaceans inhabiting cold waters, like the harbor porpoise, is organized in different regions presenting histological and functional specializations. The tail and the outer layer of the thorax blubber are thought to be structural tissues, while the inner part of the thorax blubber is more likely representing an energy source. In fact, the inner thorax blubber is preferentially lost during starvation, in contrast to the tail blubber [1,24]. In acoustic tissues, *i*-FA are thought to be concentrated in the internal melon whereas external tissues possess longer and less branched FA [12]. Acoustic fats containing mainly short and *i*-FA are thought to lower the velocity of sounds [25]. Their localization in the central part of the melon could therefore explain how this organ functions as a positive acoustic lens. Internal tissues with a low sound velocity are surrounded by higher sound velocity tissues in order to focus the sounds produced by the MLDB complex [3,6,9,25].

Analyses of the composition of cetacean adipose tissues indicated the presence of sixty to seventy different FA [10,11]. This huge heterogeneity relies on the presence of chain-length ranging from four to twenty-four carbon atoms (including not only even FA but also odd ones), on different degrees of unsaturation (from saturated FA to highly unsaturated FA like docosahexaenoic acid (22:6)) as well as on the presence of straight-, *iso*- and *anteiso*-FA. Such a complexity, together with the various factors impacting the FA composition described above, generate technical challenges for identifying and quantifying each FA molecular species. In most studies, lipids were first extracted from cetacean adipose tissues using chloroform methanol mixtures. The FA composition was then determined using GC of FABEs (fatty acid butyl esters) derivatives because of the high volability of the short-chain FA. Although this approach is powerful, it does not allow for the quantification of the fatty alcohols parts of the WE, which are not reported in most studies. A couple of studies used NMR (nuclear magnetic resonance) to globally characterize the lipid content and composition of cetacean adipose tissues [13,14,26]. If this technique does not depend on any prior extraction step, it does not result in very precise FA compositions since FA with identical structure but different chain-length cannot be resolved. In addition, because of the poor resolution of ^1^H spectra, long ^13^C NMR analyzes are required to get exploitable results. Therefore, although promising, this NMR approach still needs optimization. Surprisingly, if both approaches (GC and NMR) generated valuable information concerning the lipid and FA compositions of the adipose tissues from cetaceans, no report has so far compare their advantages and drawbacks.

In the present study, we used for the first time to our knowledge HR-MAS NMR (High Resolution Magic Angle Spinning Nuclear Magnetic Resonance) to the study of lipid composition of the adipose tissues of cetaceans. We also performed GC-based analyses, but using a procedure yielding FAMEs (Fatty Acid Methyl Esters) to also recover and analyze free fatty alcohols. We applied in parallel both techniques to characterize and quantify lipids and fatty acyl-chains from two odontocetes species, the harbour porpoise (*Phocoena phocoena*) and the long-finned pilot whale (*Globicephala melas*). Samples from blubber and melon were taken at internal and external positions, and analyzed directly by HR-MAS NMR as well as by classical GC after lipid extraction. The generated compositions were compared, on both qualitative and quantitative aspects, in order to evaluate the quality and precision of each technical pipeline. Our results show that these two approaches are very complementary and collectively allow for generating lipid compositions of very high quality.

## Materials and methods

### Chemicals

All chemicals, solvents, and lipid standards/references were purchased from Merck (Darmstadt, Germany) or Sigma (St Louis, MO, USA).

### Origin of samples

Samples were taken from fresh carcasses of recently stranded animals provided by the French Stranding Network (described in [27]). Entire melons and dorsal blubber fragments, taken behind the dorsal fin, were collected either directly on the stranding site, or during a necropsy, and kept frozen at -30°C until use. In the laboratory, subsamples of 20 mm long, 5 mm wide and 5 mm thick were taken from frozen blubbers or melons, bisected, and each half was sent frozen to one of the analysis platforms (HR-MAS NMR and GC).

### HR-MAS NMR analysis

HR-MAS NMR spectra were acquired on an AVANCE III HD 500 MHz spectrometer (BrukerBioSpin, Wissembourg, France) equipped with an indirect HR-MAS ^1^H/^31^P probe head with Z-gradient. A piece of tissue (about 10 mg) was placed in a 4 mm zirconium oxide (ZrO_2_) MAS rotor and D_2_O was added into the rotor for ^2^H field locking. The rotor was then placed under spinning (spinning rate of 5000 Hz) at the so-called “magic angle” of 54.7° with respect to the magnetic field B_0_. Before each experiment, parameters were optimized: pulse calibration at PLW1 = 20W was determined and manual shimming was performed to obtain linewidth under 7 Hz. ^1^H HR-MAS NMR spectra were performed, at room temperature, with a 30° pulse, a 2 s delay, 64 scans (spectral width = 10 000 Hz, TD = 32 K and SI = 64 K) using a Watergate sequence. Longer delays were tested but integration values did not change indicating a total relaxation of all spectral protons. Fourier transformation was performed with exponential multiplication (line broadening = 0.3 Hz). Each spectrum was then phased and baseline-corrected using a polynomial function. Spectra were calibrated using trimethylsilyl propionate as reference compound. Integration of the different peaks was performed to allow the quantification of seven different parameters described in the results section. As reproducibility test, three sub-samples were independently analyzed three times each, and manual integrations gave an error below 5%.

### Quantitative analysis by HR-MAS NMR

For the quantitative analysis of HR-MAS NMR spectra, an arbitrary value of 1000 was first assigned to the area of the well-resolved peak *q*. The area values of the other peaks was measured proportionally, either directly or using simple correlations as described in the result section. Taking into account the number of hydrogen(s) of the corresponding functional groups, these values were then used to determine the relative proportions of the two classes of neutral lipids, i.e. TAG (triacylglycerides) and WE (wax esters), and the relative proportions of five classes of fatty acids (long linear FA, long iso-branched FA, ω3-FA, IVA and IBA + 2-MBA) using the following equations:

- Proportion of TAG = A(*o*) / (A(*o)* + A(*w*))
- Proportion of WE = A(*w*) / (A(*o)* + A(*w*))
- Proportion of long linear FA = Â(*a*) / (Â(*a)* + Â(*b*)/2 + Â(*c*) + A(*d*,*x*)/2 + Â(s)/2)
- Proportion of long iso-branched FA = (Â(*s*)/2) / (Â(*a)* + Â(*b*)/2 + Â(*c*) + A(*d*,*x*)/2 + Â(s)/2)
- Proportion of ω3-FA = Â(c) / (Â(*a)* + Â(*b*)/2 + Â(*c*) + A(*d*,*x*)/2 + Â(s)/2)
- Proportion of IVA = (Â(*b*)/2) / (Â(*a)* + Â(*b*)/2 + Â(*c*) + A(*d*,*x*)/2 + Â(s)/2)
- Proportion of IBA + 2-MBA = (A(*d*,*x*)/2) / (Â(*a)* + Â(*b*)/2 + Â(*c*) + A(*d*,*x*)/2 + Â(s)/2)

In the formula, symbol A(*z*) corresponds to the measured area of peak *z*, A(*z*,*y*) to the measured area of peaks *z* and *y*, and Â(z) to the calculated area of peak *z* (see Table 1 for details). For the relative quantification of TAG and WE, their proportions were equal to the area of their characteristic peak divided by the sum of area of two characteristic peaks. For the different types of FA, the terminal methyl was used as characteristic peak, and IBA and 2-MBA were quantified together, when both present, as peak *d* and *x* areas could not be measured separately. The relative proportion was equal to the area of the characteristic peak divided by the sum of area of the five characteristic peaks. The coefficient 2 was used for iso-branched compounds which contain two terminal methyls. It should be pointed out that following this reasoning the ratio of IBA + 2-MBA is slightly underestimated as if IBA (peak d) contain two terminal methyls, 2-MBA (peak x) has just one.

**Table 1:** Assignment of the 24 major peaks detected by HR-MAS NMR in the blubber and melon of the harbour porpoise and long-finned pilot whale, and calculations used to determine peak area.

Peak	^1^H δ(ppm)	Assignment		Component moiety	Area value
a	0.87	Terminal methyl function	C**H**_3_	long linear FA chains (except ω3)	= α(a,s)—α(s) = A(a,s)—Â(s) = A(a,s) - 3*A(t)
b	0.93 (d, ^3^J_HH_ = 6.6 Hz)	Isopropyl methyl	(C**H**_3_)_2_-CH-CH_2_-CO-O-	IVA chain	= 3*(α(j) + α(v)) = 3*(A(j) + A(v))
c	0.94 (t, ^3^J_HH_ = 7.5 Hz)	Terminal methyl function	C**H**_3_-CH_2_-CH =	ω3 chains	= α(b,c)—α(b) = A(b,c)—Â(b) = A(b,c)—(3*(A(j) + A(v)))
d	1.13	Isopropyl methyl	(C**H**_3_)_2_-CH-CO-O-	IBA chain	measured directly (but impossible to separate from peak x)
e	1.26–1.32	Methylene	-(C**H**_2_)_n_-	All FA chains	measured directly
f	1.53	β-methylene to ester function	-C**H**_2_-CH_2_-CO-O-	All FA chains	= α(f,u)—α(u) = A(f,u)—Â(u) = A(f,u)—(A(t)/2)
g	2.02	α-methylene to double bond	-C**H**_2_-CH = CH-	MUFA and PUFA chains	= α(g,h,i)—α(h)—α(i) = A(g,h,i)—Â(h)—Â(i) = A(g,h,i)—(2/3*Â(c))—Â(i) = A(g,h,i) - 2/3* (A(b,c)—(3* (A(j) + A(v)))) —(A(j) + A(v))/2
h	2.05	α-methylene to double bond and terminal methyl function	CH_3_-C**H**_2_-CH = CH-	ω3 chains	= 2/3 α(c) = 2/3*(A(b,c)—(3*(A(j) + A(v)))
i	2.08 (m, ^3^J_HH_ = 6.7 Hz)	Methine	(CH_3_)_2_-C**H**-CH_2_-	IVA chain	= (α(j) + α(v))/2 = (A(j) + A(v)) /2
j	2.15 (d, ^3^J_HH_ = 5.8 Hz)	α-methylene to ester function	(CH_3_)_2_-CH-C**H**_2_-CO-O-	IVA chain on glycerol	measured directly
k	2.25	α-methylene to ester function	-CH_2_-C**H**_2_-CO-O-	All FA chains	measured directly
l	2.30	α-methylene to ester function and double bond	CH = CH-C**H**_2_-CO-O-	MUFA and PUFA chains	measured directly
m	2.50	α-methine to ester function	(CH_3_)_2_-C**H**-CO-O-	IBA chain	measured directly (but often too weak for correct integration)
n	2.77–2.83	Polyunsaturated methylene	-CH = CH-C**H**_2_-CH = CH-	PUFA chains	measured directly
o, p	4.07, 4.26	*sn*-1 and *sn*-3 esterified glycerol	-C**H**_2_-O-	Glycerol	measured directly
q	5.20	*sn*-2 esterified glycerol	-OCH_2_-C**H**(-O-)-CH_2_O-	Glycerol	measured directly
r	5.30–5.33	Olefinic hydrogens	-C**H** = C**H**-	MUFA and PUFA chains	measured directly
s	0.86 (d, ^3^J_HH_ = 6.7 Hz)	Isopropyl methyl	(C**H**_3_)_2_-CH-CH_2_-(CH_2_)_n_-	isobranched long chains	= 3*α(t) = 3*A(t)
t	1.15	Methylene	(CH_3_)_2_-CH-C**H**_**2**_-(CH_2_)_n_-	isobranched long chains	measured directly
u	1.51 (m, ^3^J_HH_ = 6.6 Hz)	Methine	(CH_3_)_2_-C**H**-CH_2_-(CH_2_)_n_-	isobranched long chains	= α(t)/2 = A(t)/2
v	2.17 (d, ^3^J_HH_ = 6.9 Hz)	α-methylene to ester function	(CH_3_)_2_-CH-C**H**_2_-CO-O-	IVA chain on WE	measured directly
w	4.00	α-methylene to ester function	-CO-O-C**H**_2_-	WE	measured directly
x	1.12	Methyl group	CH_3_-CH_2_-CH(C**H**_**3**_)-CO-O-	2-MBA	measured directly (but impossible to separate from peak d)

### Lipid extraction

A piece of tissue (about 20 mg FW (Fresh Weight)) was homogenized with a Polytron (5 times 30 s at 20 000 rpm, with keeping samples on ice for one minute between each disruption) in 2 ml chloroform/methanol (1:2, v/v), and the lipids were extracted for 4 hours at 4°C under shaking. The organic phase was recovered and lipids were further extracted from the residue with successively 1.5 ml of chloroform/methanol (1:1, v/v) and 1 ml of chloroform. The organic phases were combined, extracted with 2 ml of 2.5% NaCl (w/v) and dried on hydrophilic cotton. After evaporation under a gentle stream of nitrogen, the lipid extracts were dissolved in chloroform/methanol (1:1, v/v) at a final concentration of 5 mg FW/ml, and stored at 4°C until used.

### Fatty acyl-chain analysis by gas chromatography / mass spectrometry

Fatty acyl-chains were released by transmethylating 50 μl of the lipid extracts (equivalent to about 250 μg FW) for 1 hour at 85°C in 1 ml of methanol containing 0.5 M sulphuric acid and 2% (v/v) dimethoxypropane. After cooling, 1 ml of NaCl (2.5%, w/v) and 500 μl of hexane were added to extract FAMEs and free fatty alcohols. Following phase separation by centrifugation, the organic phases were transferred to sealed vials and directly analyzed by gas chromatography analysis using an Agilent 7890A gas chromatograph equipped with an HP-5 column (30 m x 0.32 mm x 0.25 μm) and a flame ionization detector. The initial temperature of 33°C was held for 5 min, increased at 25°C/min to 150°C, further increased at 10°C/min to 320°C and held for 3 min at 320°C, using helium (1.5 ml/min) as carrier gas.

The different acyl-chains were identified by comparison with appropriate reference substances and by mass spectrometry using an Agilent 6850 gas chromatograph equipped with an HP-5MS column (30 m x 0.25 mm x 0.25 **μ**m) and an Agilent 5975 mass spectrometric detector (70 eV, mass to charge ratio 50 to 750) using the same program. In addition, the chain length of the free fatty alcohols was confirmed after silylation of the free hydroxyl groups. For that purpose, the hexane phase containing the FAMEs and the free fatty alcohols was dried under a gentle stream of nitrogen, dissolved into 100 **μ**l of BSTFA-TMCS (*N*,*O*-bis(trimethylsilyl)trifluoroacetamide:trimethylchlorosilane; 99:1), and free hydroxyl groups derivatized at 110°C for 15 min. After evaporation of the surplus of BSTFA-TMCS under nitrogen, samples were dissolved in 500 **μ**l hexane and analyzed by GC-MS using the same program. Finally, the nature of the polyunsaturated fatty acids was confirmed by injection on a polar column using an Agilent 7890A gas chromatograph equipped with a DB-Wax column (30 m x 0.53 mm x 1 **μ**m) and a flame ionization detector, and by comparison with the retention times of standards. The initial temperature of 150°C was held for 1 min, increased at 5°C/min to 240°C and held for 5 min at 240°C, using helium (1.5 ml/min) as carrier gas.

### Wax ester purification and profiling

Lipid extracts were first separated by preparative TLC (Thin Layer Chromatography) using silica gel 60 plates (Merck, New York, USA) and hexane/diethyl ether/acetic acid (30/5/0.66 (v/v/v)) as solvent system. WE and TAG were visualized using 0.01% [w/v] primuline in acetone:water (8:2 (v/v)) and appropriate standards, and scrapped off the plate with a razor blade. TAG were directly transmethylated and their fatty acid composition determined by GC as described above. WE were extracted from the silica as described in Domergue *et al*., [28], resuspended in a small volume of hexane and analyzed by GC as described above. In some case, the identity of the medium and long-chain fatty acyl components of WE was confirmed by transmethylation, silylation and analyzed by GC as described above.

## Results

### Preparation of samples

We selected in the present study two species of odontocetes, a phocoenid, the harbour porpoise (*Phocoena phocoena*) and a delphinid, the long-finned pilot whale (*Globicephala melas*), from which we prepared eight different samples. These samples were taken from their two major adipose tissues, the blubber, at outer and inner positions of the thorax, and the melon, at central and external positions (S1 Table). The "outer blubber" was collected five millimeters under the epidermis, whereas the "inner blubber" was collected in the deep layer, five millimeters above the muscle. For the melons, the "central melon" was taken in the center of the organ, whereas the "external melon" was taken near the epidermis. Each of these samples was then bisected, and each half used for lipid analysis by GC-MS or HR-MAS NMR.

### Fatty acyl chain composition analysis by gas chromatography

**Fig 1:**
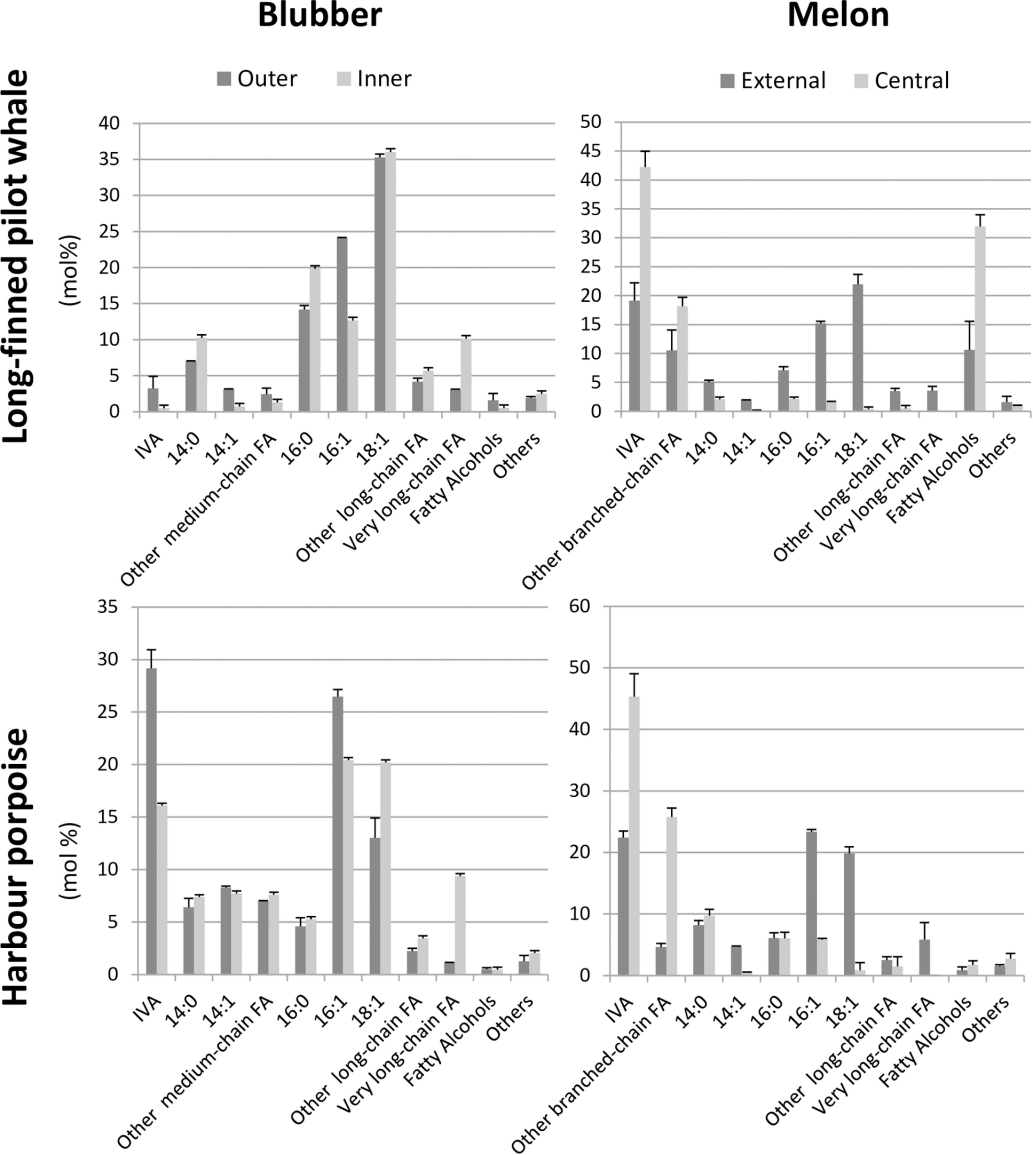
Fatty acyl compositions of the blubber and melon samples from harbor porpoise and long-finned pilot whales obtained by gas chromatography. Acyl-chains were released by acid-catalysed transmethylation, separated by GC and detected by flame ionization detector (FID). The major fatty acids and fatty acyl classes are reported (see S2 Table for exact acyl chain compositions). Amounts are given in mol% and each value is the mean +/- SD from at least three replicates.

Lipid extracts were prepared from all samples and aliquots equivalent to about 250 **μ**g FW directly transmethylated in order to determine their fatty acyl composition by gas chromatography. This procedure allowed for the quantification of both FA (in the form of FAMEs) and fatty alcohols (Fig 1; S2 Table). In order to reduce as much as possible the loss of short-chain FA, which are highly volatile and partially soluble in water, our procedure was devoid of any aqueous extraction and solvent removal step.

The long-finned pilot whale (hereafter referred to as LFPW) blubber samples were dominated by long-chain (16:0, 16:1 and 18:1) and medium-chain (C12 to C15) FA, which altogether represented about 80% of the total acyl-chains (Fig 1). Harbour porpoise (hereafter referred to as HP) blubber samples were rich in the same long-chain FA, but contained more medium-chain FA (12:0, 12:1, 13:0 and 14:1) as well as high amounts of IVA, which represented up to 30 and 17% of the total in the outer and inner blubber, respectively. In both species, the fatty alcohols levels were low, and the inner blubber was enriched in very long-chain fatty acids (including the ω-3 eicosapentaenoic and docosahexaenoic acids) when compared to the outer blubber. Inversely, the outer blubber of both species was enriched in IVA acid when compared to the inner blubber.

In both species, the external melons were dominated by IVA and the long-chain monounsaturated FA 16:1 and 18:1, each accounting for about 20% of the total (Fig 1). In contrast, central melons contained much less 16:1 and 18:1, and were highly enriched in IVA, which represented almost 50% of the total. Central melons also contained high levels of branched-chain FA, especially iso-pentadecanoic acid (*i*-15:0; S2 Table). In addition, very long-chain FA (including the ω-3 eicosapentaenoic and docosahexaenoic acids) were present in external melons, but undetected in central melons. LFPW and HP melons mainly differed in that the former contained high amounts of fatty alcohols, especially branched-chain (iso) fatty alcohols, whereas the latter contained very few of these. In both species, the fatty alcohol levels were higher in the central melon than in the external melon.

### HR-MAS NMR spectra analysis of blubber and melon samples: Triacylglycerols, wax esters and fatty acid classes

**Fig 2:**
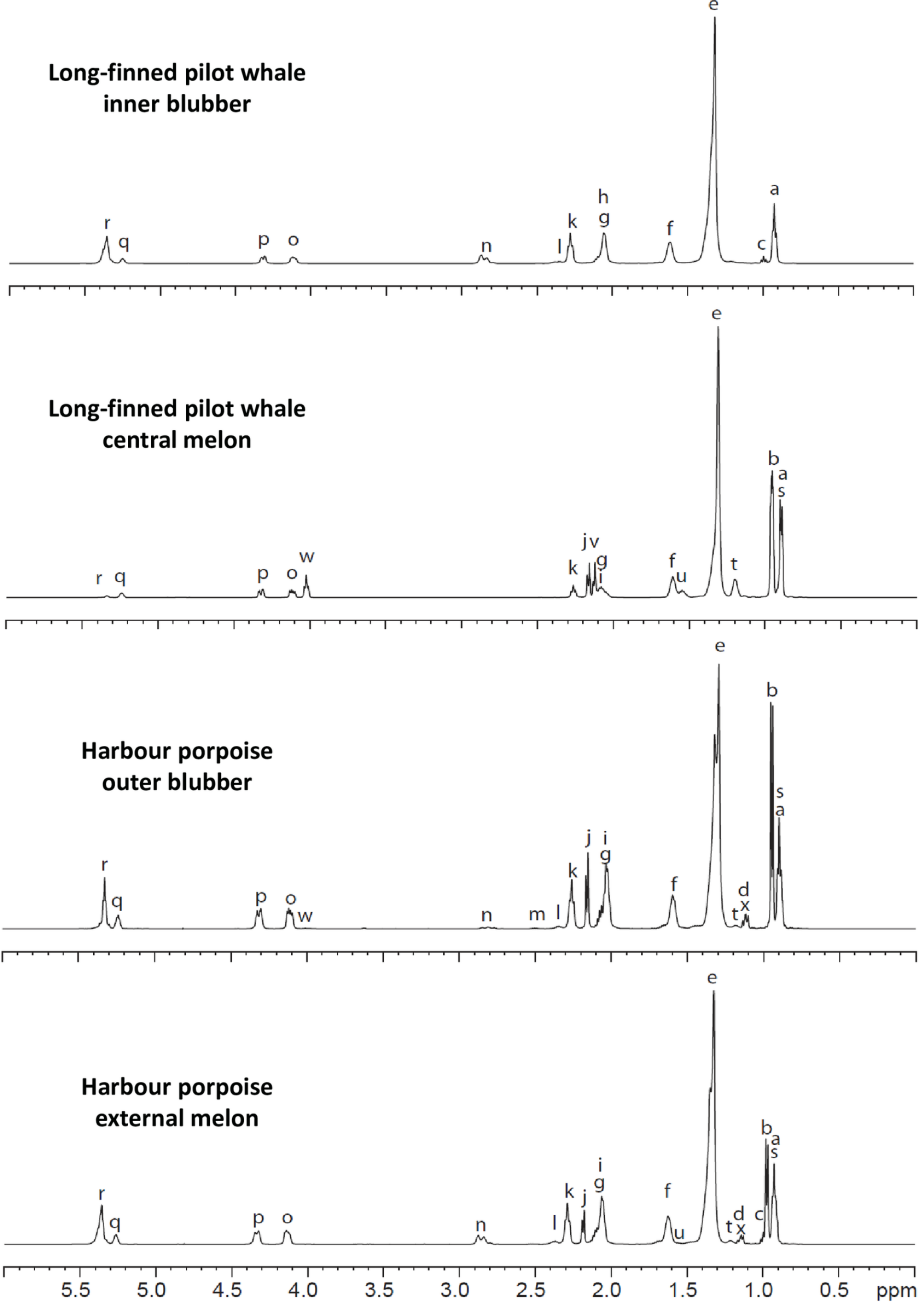
Representative ^1^H HR-MAS spectra of the samples. Intact tissues were placed in a zirconium oxide MAS rotor, D_2_O was added for ^2^H field locking and ^1^H HR-MAS NMR spectra were acquired at room temperature (spinning speed = 5000 Hz and ns = 64). The assignment of peaks *a* to *x* is given in Table 1.

The same eight samples were in parallel analyzed using HR-MAS NMR and the spectra obtained are presented in Figs 2 and S1. All HR-MAS NMR spectra showed a high degree of resolution with several individual peaks characteristic of distinct lipid classes presenting no overlap. In total, 24 peaks were unambiguously characterized on the basis of their chemical shift (between 0.86 and 5.33 ppm) and multiplicity. The first 18 major peaks were named from “*a*” to “*r*” in alphabetical order following increasing shifts on the first samples analyzed. Six further peaks, minor in quantity, were named from "s" to "x" in the order of their characterization in subsequent analyzes. The identification of all these 24 peaks, which was confirmed using 2D HR-MAS NMR correlation spectroscopy, is presented in Table 1. All peaks corresponded to different parts of the two major lipid classes found in cetacean adipose tissues, i.e. TAG and WE. No signals were detected for chemical shifts superior to 5.33 ppm, excepted a very weak one around 11 ppm corresponding to carboxylic acid function, indicating that only very few free fatty acids (FA) were present in the samples.

Since the adipose tissues we analyzed mainly contained TAG and/or WE, both of them being made of fatty acyl-chains, methylene (–CH_2_–) and methine (–CH =) groups accounted as expected for most of the major of peaks (13 out of 24) between 1.12 to 2.83 ppm (i.e peaks *e-n*, *t*, *u* and *v*, Table 1). These different shifts reflected the proximity of the group (i) to a double bond, (ii) to a branch point or (iii) to an ester function (Table 1). Similarly, methyl groups (CH_3_–) gave six signals (*a-d*, *s* and *x*, between 0.86 to 1.13 ppm) according to their chemical environment (Table 1). In addition, the olefinic hydrogen signal, typical of unsaturation, was detected at 5.3 ppm (peak *r*; Table 1). Finally, the glycerol backbone produced 3 clear signals, *o*, *p* and *q*, respectively at 4.07, 4.26 and 5.20 ppm, while one signal, (*w*) at 4.00 ppm, was specific to WE (Table 1).

Although HR-MAS NMR analysis cannot identify all the different acyl chains present in the samples, several classes of FA as well as some specific fatty acids could be unambiguously identified (i) by their terminal methyl function, (ii) by the adjacent methine functions or (iii) by methylene function adjacent to ester functions (Table 1). Long linear FA presented one specific peak (*a*), long iso-branched FA three specific peaks (*s*, *t*, and *u*) and ω3-FA two specific peaks (*c* and *h*). More importantly, IVA and IBA were unambiguously characterized by, respectively, three (*b*, *i*, *j* or *v*) and two (*d* and *m*) peaks. In addition, 2-MBA could be identified with one specific peak (*x*).

### Principles and reproducibility of a quantitative analysis by HR-MAS NMR

As the linewidth of the ^1^H HR-MAS NMR spectra we obtained was high enough to discriminate between resonances that were close in frequency, the high resolving power of HR-MAS NMR prompted us to perform quantitative analyses of the spectra. The area of the well-resolved peak *q* (*sn-2* position of esterified glycerol, Table 1) was assigned an arbitrary value of 1000, and area values of the other peaks were determined proportionally. Out of the 24 peaks, 15 (including *q*) had their area values directly measured from the spectra (Table 1). Despite the high spectral resolution, some peaks were nevertheless very close, sometimes overlapping, not allowing their direct measurements. For instance, peak *b* (IVA chains) and *c* (ω3 chains) had chemical shifts of 0.93 and 0.94 ppm, respectively (Table 1). It was nevertheless possible to distinguish one from the other by their multiplicity: the isopropyl methyl of IVA appeared as a doublet whereas the terminal methyl of ω3 FA appeared as a triplet. For instance, the LFPH inner blubber contained ω3-FA (peak *c*) and no detectable IVA (peak *b*), whereas the HP external melon contained a lot of IVA, but very little ω3-FA (Fig 2). Similarly, peak *s* (0.86 ppm) and *a* (0.87 ppm) overlapped but could be distinguished because the former appeared as a doublet whereas the latter as a triplet. However, all peaks could not be distinguished one from another. For example, peaks *g*, *h* and *i*, were all around 2.05 ppm and appeared all as multiplets. For calculating their area, we used the fact that several compounds produce different peaks, the area of which being proportional to the number of hydrogen detected. For example, long-chain isobranched FA were detected by three different peaks (*s*, *t* and *u*) and the area of peak *s* (6 hydrogens) had to be three times that of peak *t* (2 hydrogens) and six times that of peak *u* (one hydrogen). We therefore took advantage of such correlations to calculate the area of 2 compounds present in overlapping peaks. If peaks *a* and *s* overlapped, the area of peak *s* could be deduced from the well-resolved peak *t*, and that of peak *a* calculated by a simple subtraction (see Table 1). Following this reasoning, and taking most importantly into account the number of hydrogen atoms of each particular chemical function, we could evaluated the areas of 22 out of the 24 peaks. Details of the calculation for each signal are presented in Table 1. It should be pointed out that since the areas of peaks *d* and *x* could not be measured separately, and as peak *m* was often too low for being correctly integrated, only the sum of IBA and 2-MBA could be estimated by HR-MAS NMR.

We then used these values to determine seven quantitative parameters for each spectrum. These parameters correspond to relative proportions between two or more area values, corrected by coefficients depending on the number of hydrogen(s) of the corresponding functional groups as indicated in the Materials and Methods section. These parameters are the proportions of WE (represented by the area of the peak *w*) and TAG (peak *o*) relative to the sum of TAG and WE, and the proportions of long linear FA (peak *a*), long *i*-FA (peak *s*), ω3-FA (peak *c*), IVA (peak *b*), and IBA and 2-MBA (sum of the peaks *d* and *x*) relative to the sum of the all five classes. All these relative proportions were calculated for each sample as the ratio between the characteristic peak(s) and the sum of all peaks taken in consideration. When 3 samples (HP inner and outer blubbers, and LFPW central melon) were quantitatively analyzed in 3 independent experiments, these parameters were nearly the same for each triplicate analysis (S2 Fig), supporting the reproducibility of the HR-MAS NMR method.

### Characterization of the overall lipid compositions by HR-MAS NMR

**Fig 3:**
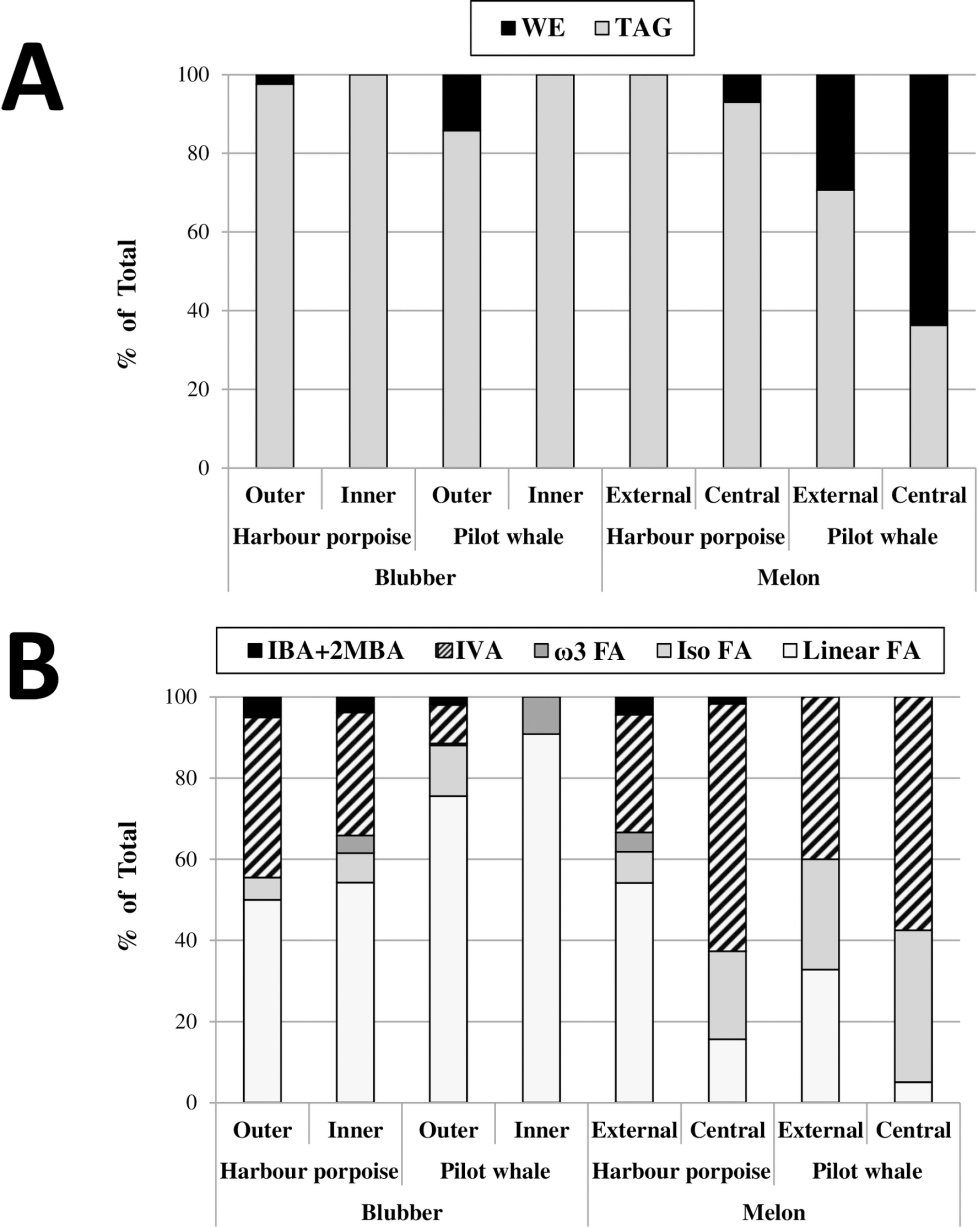
Overall lipid compositions according to HR-MAS NMR analyses. Class of neutral lipids (A) and types of fatty acyl-chains (B) were quantified and compared between the 8 samples according to HR-MAS NMR analyses. Amounts are given in % of total. IBA+2MBA, relative amount of isobutyric and 2-methylbutyric acids; IVA, relative amount of isovaleric acid; ω3 FA, relative amount of omega3-fatty acids; Iso FA, relative amount of isobranched fatty acyl-chains; Linear FA, relative amount of linear fatty acyl-chains.

The overall lipid composition of the eight samples analyzed by HR-MAS NMR (Fig 3) was then determined using the method and parameters described above. As shown in Fig 3A, most of the samples were dominated by TAG, with the exception of the LFPW central melon, which contained more WE than TAG. WE were systematically more abundant in tissues from the LFPW than in the corresponding ones from the HP (Fig 3A). Concerning the different FA classes, blubbers were dominated by long-chain linear FA whereas melons contained high amounts of IVA and *i-*FA (Fig 3B). IVA represented about 60% of all FA in both central melons and as much as 30 and 40% in the external melons from the HP and the LFPW, respectively. High levels of IVA were also detected in the HP blubber, whereas it was minor in outer and absent in inner blubber samples from the LFPW, respectively. Long *i*-FA were observed in all samples except in the inner blubber of the LFPW. They were particularly abundant in the central melon of the HP as well as in both melon samples from the LFPW (Fig 3B). IBA and 2-MBA were observed in small proportions in the four samples of the HP, but only detected in the outer blubber of the LFPW. Finally, the level of ω3-FA was very low in all samples except for the LFPW inner blubber and the HP inner blubber and external melon, where it represented 5 to 10% of the total FA.

When comparing the composition of each tissue within the species, the inner blubber had generally higher proportions of long-chain FA and ω3-FA whereas the outer blubber was enriched in short-chain FA (IVA, IBA and 2-MBA). Concerning melons, the central melon had higher proportions of branched-chain FA (IVA and long-chain *i*-FA), and lower proportions of linear long-chain FA than the external. It can be noted that if the overall FA composition of the central melon from both species were rather similar, the composition of external melon clearly differed. In the case of the HP, the composition of the external melon was surprisingly very close to that of the inner blubber. In contrast, the inner and outer melons from the LFPW presented a rather similar composition.

### Comparison of the HR-MAS NMR and GC lipid compositions

In order to compare the overall lipid compositions determined by the 2 techniques, the results obtained by GC analysis were then processed to determine the same 7 quantitative parameters obtained through HR-MAS NMR analysis. As each WE molecule is made of one fatty acid esterified to one fatty alcohol, the content of acyl-chains in WE was calculated by doubling the total amount of fatty alcohols. Following the same reasoning, the content of acyl-chains in TAG corresponded to total amount of fatty acids minus the total amount of fatty alcohols. Concerning the five different FA classes, their relative proportions were easily quantified by GC since this technique allowed the separation and clear identification of all acyl-chains (S2 Table). It should be noted that for linear and *i-*FA, values accounted for all fatty acyl classes of this type (i.e. for fatty acids plus fatty alcohols), since signals from the terminal methyl group (peak “a” and “s”, respectively) were used for the NMR-based quantification of these FA classes.

**Fig 4:**
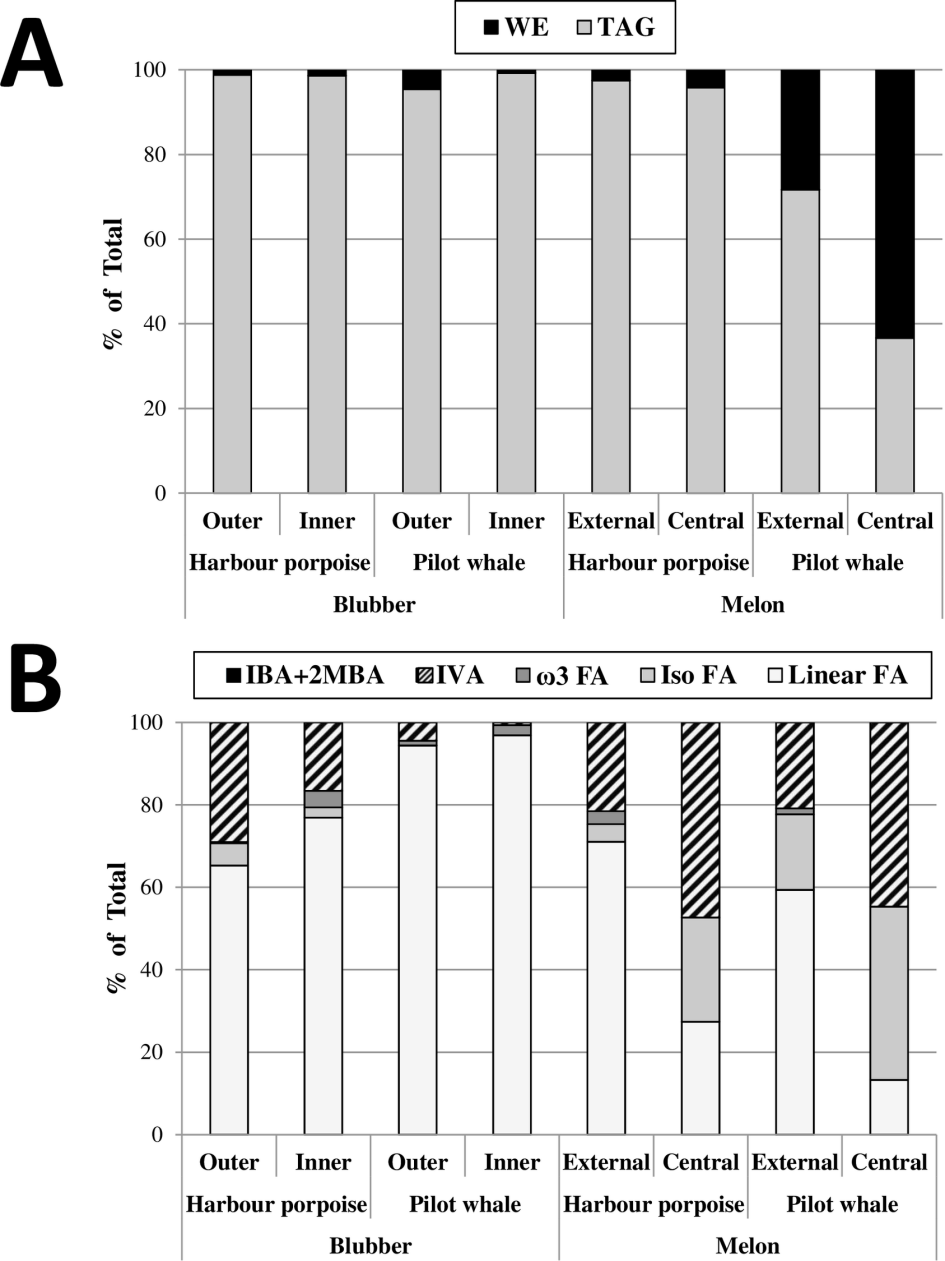
Overall lipid compositions according to GC analyses. Class of neutral lipids (A) and types of fatty acyl-chains (B) were quantified and compared between the 8 samples according to GC analyses. Amounts are given in % of total. IBA+2MBA, relative amount of isobutyric and 2-methylbutyric acids; IVA, relative amount of isovaleric acid; ω3 FA, relative amount of omega3-fatty acids; Iso FA, relative amount of isobranched fatty acyl-chains; Linear FA, relative amount of linear fatty acyl-chains.

Fig 4A shows the relative proportions of TAG and WE as well as those of the five FA classes in the 8 different samples according to GC analyses. The TAG to WE ratios were in very good agreement with the quantitative data obtained by HR-MAS NMR analysis (compare Figs 3A and 4A), especially for the two tissues containing the highest WE contents (LFPW external and central melons). Nevertheless, when the level of WE was very low, a difference between results obtained by GC and HR-MAS NMR was observed, as HR-MAS NMR failed to detect WE in three samples (inner blubber of HP and LFPW, and external melon of HP).

The relative proportions of the five FA classes according to GC analysis were also in good agreement with the quantitative data obtained by HR-MAS NMR analysis (compare Figs 3B and 4B), however there were some clear differences between the results obtained by each technique, particularly concerning the quantification of the short-chain fatty acids. Two compounds, IBA and 2-MBA, were only detected by the HR-MAS NMR analysis, while GC analysis apparently systematically underestimated the IVA content. These differences are most probably due to the high volatility and partial solubility in aqueous solutions of short-chain fatty acids, which may be partially lost during the preparation of the FAMEs (see Materials and Methods). Despite this discrepancy, the relative proportions of linear long-chain and isobranched long-chain FA obtained by the two techniques were in rather good agreement. Finally, as in the case of tissues with low levels of WE, HR-MAS NMR hardly detected ω3-FA in the samples that contained very low concentrations of these. It was especially notable in the samples that contained a large quantity of IVA compared to ω3-FA, like the outer blubber of the HP or the external melon of the LFPW. Indeed, overlapping of IVA and ω3-FA signals (respectively peaks *b* and *c*) complicated the calculation of the respective integrals.

### Wax ester profiling

**Fig 5:**
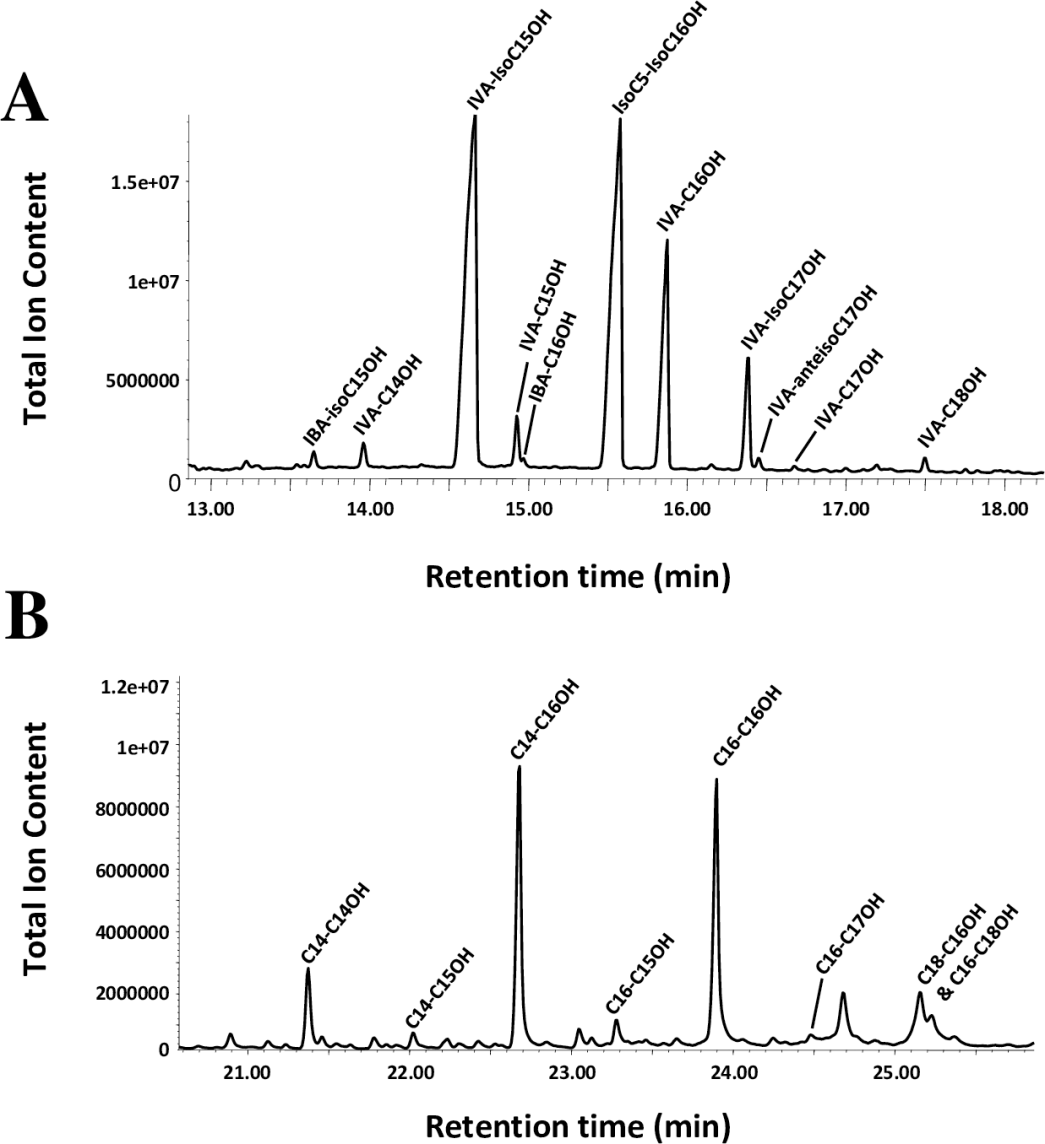
WE profiling of the central melon and outer blubber from the long-finned pilot whale. WE from the central melon (A) and outer blubber (B) were purified by TLC before analysis by GC-MS. Note the different retention time windows between panel A and B. Identification of the major WE molecular species is provided in Supplemental Fig 3.

We then purified the WE fraction of several tissues by thin layer chromatography and analyzed their composition by GC-MS. As shown in Fig 5 for the LFPW, the central melon and outer blubber had strikingly different WE profiles. The WE from the central melon were exclusively containing short-chain *i*-FA- as acyl-moiety (Fig 5A), whereas the external blubber contained longer WE that were made of C14 to C18 FA and fatty alcohols (Fig 5B). GC-MS fragmentation analysis (S3 Fig) showed that among the central melon WE, more than 97% contained IVA as FA (while the remaining had IBA as FA), while nearly 80% contained iso-branched fatty alcohols, and were therefore composed of two branched acyl-chains. In contrast, WE from the blubber were exclusively made of straight acyl-chains.

**Fig 6:**
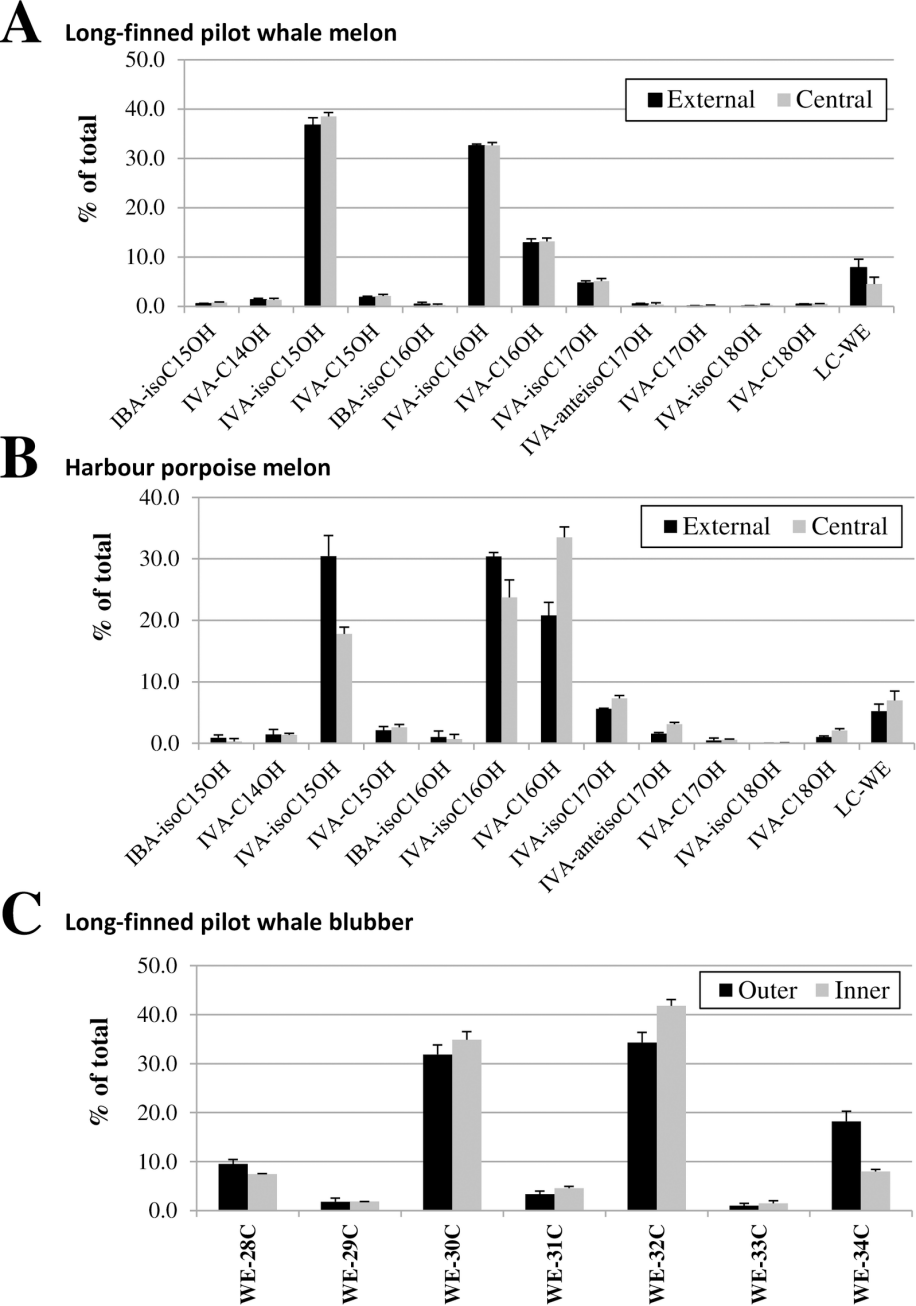
**WE profile of the melon from the long-finned pilot whale (A), the melon from the Harbour porpoise (B) and the blubber from the long-finned pilot whale (C).** WE were purified by TLC before analysis by GC-MS. Errors bars represent the variations resulting from 3 to 5 preparative TLCs used for the isolation of the WE fraction.

**Fig 7:**
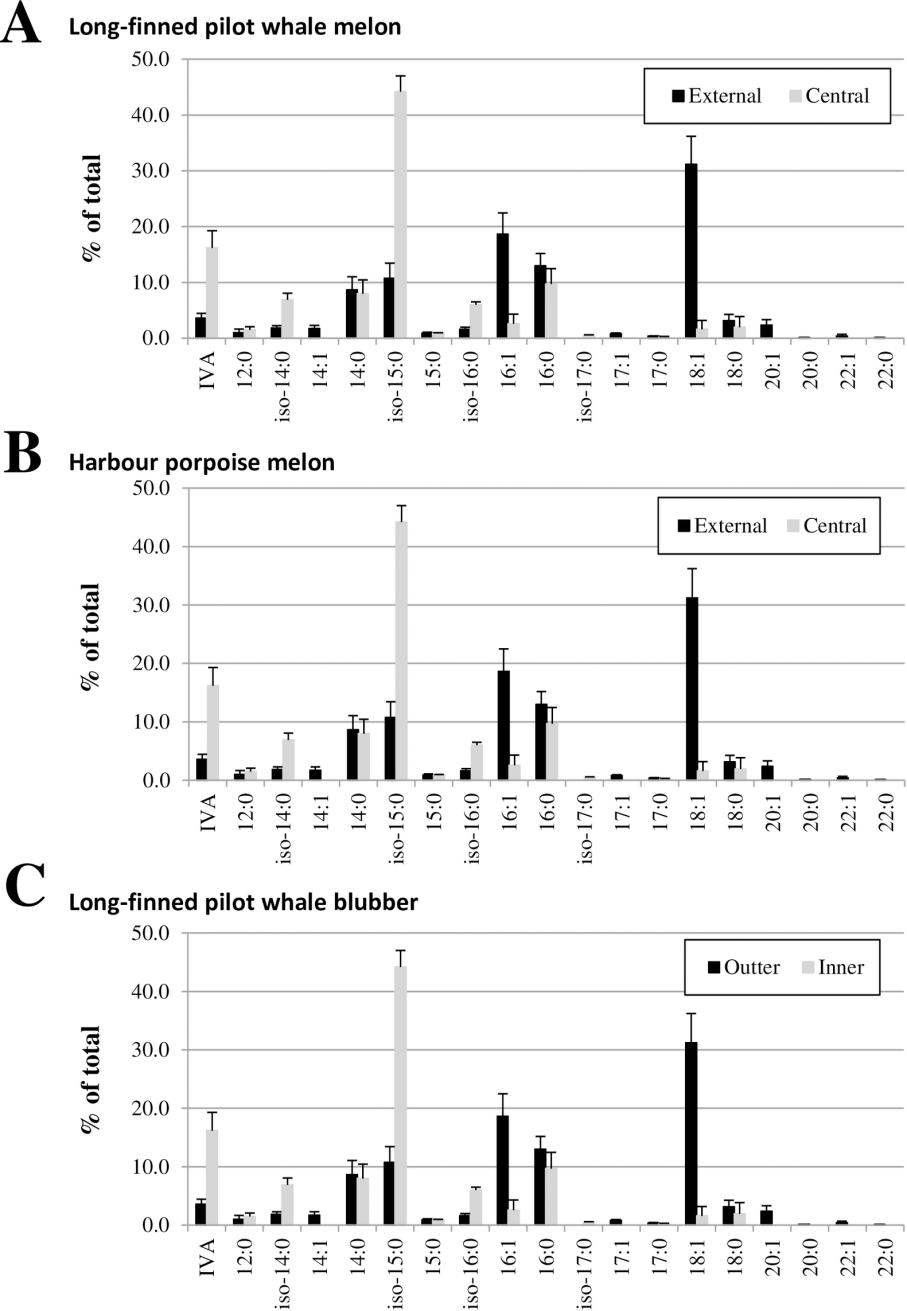
**Fatty acid composition of TAG from the melon of the long-finned pilot whale (A), the melon of the Harbour porpoise (B) and the blubber of the long-finned pilot whale (C).** TAG were purified by TLC, transmethylated and their fatty acyl composition determined by GC-MS. Errors bars represent the variations resulting from 3 to 5 preparative TLCs used for the isolation of the TAG fraction.

When comparing the different layers of the same tissue, it appeared that central and external melons had nearly identical WE compositions (Fig 6A), suggesting that the differences in total fatty acid compositions we observed (Figs 1, 4 and 5) are due to other lipids. We therefore determined the FA composition of the other main neutral lipid species of these layers, i.e. TAG, after purification by thin layer chromatography and transmethylation. As shown in Fig 7A, the FA compositions of TAGs from central and external melons strongly differed, confirming that TAG rather than WE are responsible for the different FA compositions observed in the different layers of the LFPW melon. In the HP melon, WE were similarly dominated by IVA-alkyl esters, and external and central layers had rather similar WE compositions even though the central melon was enriched in IVA-IsoC16OH WE, while the external melon contained more IVA-IsoC15OH WE (Fig 6B). Like in the LFPW melon, the TAG composition accounted for most of the difference observed in the FA composition of central and external melon (Figs 7B and 1). Similarly, the inner and outer blubber of the LFPW had very similar WE compositions (Fig 6C) while those of their TAG, which represented by far the major lipid class (Figs 4 and 5), clearly differed (Fig 7C).

## Discussion

The adipose tissues of odontocetes contain two types of unusual lipids not accumulated by other mammals. First, *i*-FA are commonly synthesized by odontocetes. Dephinidae, monodontidae and phocoenidae accumulate short *i*-FA like IVA, while other odontocetes contain longer *i-*FA, the ziphiidae accumulating for example C10-C12 *i-*FA [10,20]. Many previous studies [3–6,8,9,12–14] reported that the presence of i-FA may coincide with the acoustic properties of the melon and mandibular fats. Nevertheless, these fatty acids also found in the blubber, a tissue with no demonstrated role in sound propagation, still raising questions about their exact functions in this tissue. Second, although WE are commonly found in marine organisms [29], among mammals, only some odontocetes accumulate them [2]. Similarly, whilst most mammals cannot metabolize WE, some cetacean species feeding on WE producing preys are able to utilize WE [30]. Since TAG are thought to be more efficiently used by mammals as metabolic energy source, WE may have other advantages. For example, a correlation between WE accumulation in the blubber and the capacity to deep diving has been proposed for some odontocetes species not relying on blubber as energy source during fasting [2].

Because of its direct implication in the adaptation to the marine environment, the lipid composition of cetacean adipose tissues, and in particular the accumulation of WE and IVA, has been a subject of interest for decades [31]. Since this pioneering work, lipids and FA accumulation in different cetacean species, in different tissues and in different layers within the same tissue has been carefully described [2,5,6,9–13,19,20,23]. Nevertheless, the high complexity of the lists of lipids characterized indicated that the analytical approaches can still be improved, first by simplifying and shortening the experimental procedures to allow the analysis of numerous samples, but also concerning the difficult quantification of small (C and C5) *i*-FA [10]. In the present study, we therefore evaluated the use of HR-MAS NMR to study the global lipid composition of the blubber and melon of two odontocete species, the harbour porpoise and the long-finned pilot whale.

### Fast, easy implementation, and high spectral resolution of NMR HR-MAS

HR-MAS NMR is at the interface between liquid phase and solid state NMR. One clear advantage of HR-MAS NMR is that this type of analysis is realized on a small tissue fragment (usually 10 mg of tissue) that is directly placed in a rotor so that no prior extraction, which can affect the lipid integrity of the sample, is needed. Hence, analysis is very quick: about 20 minutes between the preparation of the sample and the spectrum display. Spinning of the rotor with a magic angle reduces the dipolar interactions within the sample. Therefore, all free molecules of the sample (i.e. TAG and WE of adipose tissues) give highly resolved NMR signals whereas phospholipids from cellular membranes, which are much less mobile molecules, produce very wide signals, which most often mix with the basal noise of the spectrum.

The analyses we performed in the present study allowed us to determine the relative proportions of WE and TAG, as well as the relative proportions of five subclasses of different FA (long linear FA, long branched (iso) FA, ω3-FA, IVA, and IBA plus 2-MBA). HR-MAS NMR provides a global view of the sample, *i*.*e*. if some compounds are major compared to others, their peak intensities will reflect these proportions. In particular, HR-MAS NMR analyses revealed unambiguous peaks showing the presence of IVA (peaks *b*, *i*, *j* and *v*), IBA (peaks *d* and *m*) as well as 2-MBA (peak *x*) in the samples. Unfortunately, since the amounts of IBA and 2-MBA were very low in all our samples, and because of overlapping signals, we considered that, even if the signal to noise ratio would be improved by increasing the number of scans, integration measurement of the weak *m* peak would generate a too high error compared to the integration of the major peaks. Thus, we only estimated the sum of IBA and 2-MBA. In contrast, analysis of identical samples in 3 independent experiments (S2 Fig) confirmed the very high reproducibility of this method. In addition, this method is non-destructive. When a sample was bisected and each subsample analyzed in parallel by GC and by HR-MAS NMR as in the present study, or when the same sample was first used for HR-MAS NMR acquisition before extraction and analysis by GC, very similar FA compositions were obtained. Any sample can therefore first be analyzed by NMR HR-MAS, and, if necessary, successively by GC.

The requirement of very small amount of tissue, the absence of extraction, the short time of analysis (about 20 minutes from sample preparation to complete acquisition for a ^1^H spectrum), the unambiguous detection of short *i*-FA, and the possibility to further process samples therefore make HR-MAS NMR a very spectacular and promising approach to study the composition and functioning of cetacean adipose tissues.

### GC-based and HR-MAS NMR-based results are corroborating and complementary

GC allowed for the separation and precise identification of the major FA present in our samples. Nevertheless our results clearly showed that, although lots of care were taken to reduce as much as possible the loss of short-chain FA (because of their high volatility and partial solubility in water), this technique systematically underestimated the real amounts of C4 and C5 FA. This limitation is a clear disadvantage when studying cetacean adipose tissues which often contain IVA (*i*-5:0) as a major constituent, and supports the use of HR-MAS NMR to correctly detect and quantify not only IVA but also IBA and 2-MBA. Very few studies have so far reported on the presence of IBA and 2-MBA in cetacean adipose tissues [8,11,13] even if the presence of longer FA derived from these precursors (like *i*-16:0 or *anteiso*-15:0, respectively) is more documented. Previous *in vivo* labeling studies using subcutaneous tissues of the striped dolphin, *Stenella coeruleoalba*, showed that IBA (and even-numbered *i*-FA) and 2-MBA (and odd-numbered *antesiso*-FA) are derived from the catabolism of branched-chain amino acids (valine and isoleucine, respectively; [16]), exactly like IVA and odd-numbered *i*-FA are derived from that of leucine [32]. Using HR-MAS NMR on melon or blubber samples from other cetacean species, we could detect IBA and 2-MBA in a high proportion of samples, suggesting that these short-chain FA are more widely spread in the adipose tissues of cetaceans than presently described in the literature.

Most studies relying on GC for determining the FA composition of cetacean adipose tissues used FABEs as derivatives in order to decrease the high volatility of short-chain FA [2,11]. In order to also quantify the fatty alcohols parts of the WE together, we decided in the present study to use FAMEs, and to reduce as much as possible the loss of short-chain FA by excluding from our procedure any aqueous extraction or solvent removal steps. This way, even though the minor IBA and 2-MBA could not be detected and IVA content was slightly underestimated, we could separate and quantify all the major FA (in the form of fatty acid methyl esters) as well as all fatty alcohols (in their free form). In addition, the identity of these later could unambiguously be confirmed by silylating the free hydroxyl groups, which impact their volatility (sharper peaks) and fragmentation (improving their identification). Therefore, whereas most studies relying on the analysis of FABEs do not report on the fatty alcohol content and composition, our FAMEs-based procedure allowed for the quantification of all fatty acyl-chains (i.e. both FA and fatty alcohols) in a single GC run.The very good correlation between the compositions we obtained by our GC- and NMR-based methods further confirmed indirectly the very low abundance of membrane lipids in adipose tissues. Whereas HR-MAS NMR allows studying “free” lipid molecules (i.e. the WE and TAG of the lipid droplets) but not those structurally bound and less mobile (i.e. the phospholipids of the membranes), GC-based methods analyze the acyl-chains from all lipids. The very similar compositions we observed in Figs 3 and 4 therefore indicated that taking in consideration the very low abundant membrane lipids (in GC analysis) or not (in NMR analysis) has nearly no impact on the total fatty acyl composition of adipose tissues.

### Non-homogenous specific lipid composition of blubbers and melons

In the present study, we used in parallel GC-based and HR-MAS NMR-based analysis to study the global lipid composition of the blubber and melon of two odontocetes, the HP and the LFPW. These two odontocetes are known to produce both WE and IVA, and to present stratified lipid compositions in their blubber as well as in their melon [2,10].

The two technical pipelines we used indicated higher proportions of WE and IVA in the outer than the inner blubber in both species, further stressing the stratified lipid compositions of this tissue. We also found that the LFPW contained more WE than the HP, especially in the outer blubber, as previously described by Koopman et al., [2]. Such difference may be related to deep diving capacity since LFPWs are known to be able to dive deep (several hundred meters deep) while the HP stays more close to the sea surface. In agreement with Koopman et al., [10], we found in contrast more IVA in the HP blubber than in the LFPW blubber. HR-MAS NMR further showed that like IVA, IBA and 2-MBA were systematically present in higher proportions in the external layer than in the inner one. Inversely, the inner layer was richer in very-long-chain FA and ω3-FA than the outer layer, in agreement with previous analyses [2,10,11]. These different compositions suggest that the inner layer, with more FA coming from the diet (i.e. ω3-FA), could be more metabolically active, whereas the outer layer, with high amounts of IVA which can be toxic, is relatively inert [11]. In addition, it may suggest that both layers have different functions, the inner one being important for energy storage, while the outer one primarily insulating and maintaining the plasticity of the body in cold waters [10].

Concerning the melon, we found in both species that the contents of WE, IVA and *i*-FA were higher in the center than in the external layer, whereas this later was enriched in C16, C18 and very-long-chain FA, as previously described [9,12,21]. In the melon, *i-*FAs are thought to be concentrated in a central area which function is to conduct and focus sounds from the MLDB complex to the external medium [6,9]. Recently, Arribart et al., [3] used magnetic resonance imaging, a technique based on the same principle as NMR, to visualize the central and external zones, most likely reflecting different lipid composition within the melon of a common dolphin. Several studies [12,21,25] already reported that sound velocity is inversely correlated with the proportion of short acyl-chain containing lipids. It is therefore conceivable that higher levels of short-chain FA and/or WE decrease neutral lipid packaging and adipose tissue density, and consequently lower sound velocity. With such an hypothesis, the different concentrations of TAG and WE containing mainly short-chain FA found in the central and external parts of the melon could explain how this organ serves as a positive acoustic lens [25]. Surrounded by the external layer, with longer and less branched FA (and therefore higher sound velocity), the center of the melon, would possibly focus the pulses generated for echolocation [12].

Our GC-based approach furthermore allowed us to identify and quantify the different WE molecular species present in the blubber and melon tissues (Figs 5 and 6). Our results clearly showed that the later was mainly composed of WE made of short chain *i*-FA (IVA, IBA and 2-MBA) and C15/C16 *iso*-fatty alcohols while the former contained only longer (≥ C28) straight-chain WE. GC-based analysis also revealed striking differences in the variations of the TAG and WE composition between the different layers of both organs (Figs 6 and 7). In both HP and LFPW, the WE composition showed very minor, if any, variations, whereas the TAG composition of the two layers analyzed clearly differed. These data therefore indicated that the stratifications of the melon and blubber were due to modifications of the WE to TAG ratio as well as to changes in the FA composition of TAG, but not to modification of the WE molecular species.

## Conclusion

Our results collectively show the increased resolving power of our dual technical approach to study the very complex composition of adipose tissues from cetaceans. HR-MAS NMR provides a global lipid composition of the sample using very little material, in a short-time frame, and without any sample preparation. It also has the clear advantage of unambiguously quantifying short-chain *i*-FAs (IVA but also IBA and 2-MBA), which represent major constituents in these tissues. GC-based analysis of FAMEs further provides the detailed acyl-chain composition of the lipid extract, as well as those of the TAG and WE fractions. This technique also allows for obtaining the WE molecular species composition.

Our approach, and especially the use HR-MAS NMR, should greatly improve our knowledge of the blubber and melon of cetaceans, which are highly structured and stratified organs. By analyzing multiple samples from a single organ, this technique could result in reconstructing biochemical topographies at a much higher level of accuracy than previously possible. In the near future, the use of NMR based analytical approach, for biochemical (this study) as well as for imaging [3] purposes, will help to further decipher the complexity of the lipid composition of odontocete adipose tissues. In particular, our understanding of the functional basis of the sound emission and reception system in odontocetes should benefit from these new improvements.

## Supporting information

S1 Fig ^1^H HR-MAS spectra of the 8 samples analyzed. Intact tissues were placed in a zirconium oxide MAS rotor, D_2_O was added for ^2^H field locking and ^1^H HR-MAS NMR spectra were acquired at room temperature. The assignment of peaks *a* to *x* is given in Table 1.
(PDF)

S2 Fig Reproducibility of the HR-MAS NMR quantitative analysis. Class of neutral lipids (A) and types of fatty acids (B) of 3 samples (harbour porpoise inner and outer blubbers, and pilot whale central melon) were quantitatively analyzed in 3 independent experiments. Amounts are given in % of total. IBA+2MBA, relative amount of isobutyric and 2-methylbutyric acids; IVA, relative amount of isovaleric acid; ω3 FA, relative amount of omega3-fatty acids; Iso FA, relative amount of isobranched fatty acids; Linear FA, relative amount of linear acids.
(PDF)

S3 Fig Mass spectra and drawing representing fragmentation of short-chain iso fatty acids-containing wax esters. **(**A) IVA-isohexadecyl ester (21Carbon-Wax ester: isoC5-isoC16OH); (B) IVA-isopentadecyl ester (20Carbon-Wax ester: isoC5-isoC15OH); (C) IBA-isopentadecyl ester (19Carbon-Wax ester: isoC4-isoC15OH); (D) IBA-hexadecyl ester (20Carbon-Wax ester: isoC4-isoC16OH).
(PDF)

S1 Table Origin of the 8 samples analyzed. (PDF)

S2 Table Fatty acyl composition of the 8 samples according to gas chromatography analyses. Amounts are given in mol% and each value is the mean +/- SD from at least three replicates. n.d., not detected.
(PDF)
