# Supplementary material for: Qualitative and quantitative study of the highly specialized lipid tissues of cetaceans using HR-MAS NMR and classical GC
Source: PLoS One. 2017 Jul 5;12(7):e0180597. doi: 10.1371/journal.pone.0180597 (PMC5498043; doi:10.1371/journal.pone.0180597)
Supplement: S1 Fig — Intact tissues were placed in a zirconium oxide MAS rotor, D2O was added for 2H field locking and 1H HR-MAS NMR spectra were acquired at room temperature. The assignment of peaks a to x is given in Table 1. (PDF) [file pone.0180597.s001.pdf]

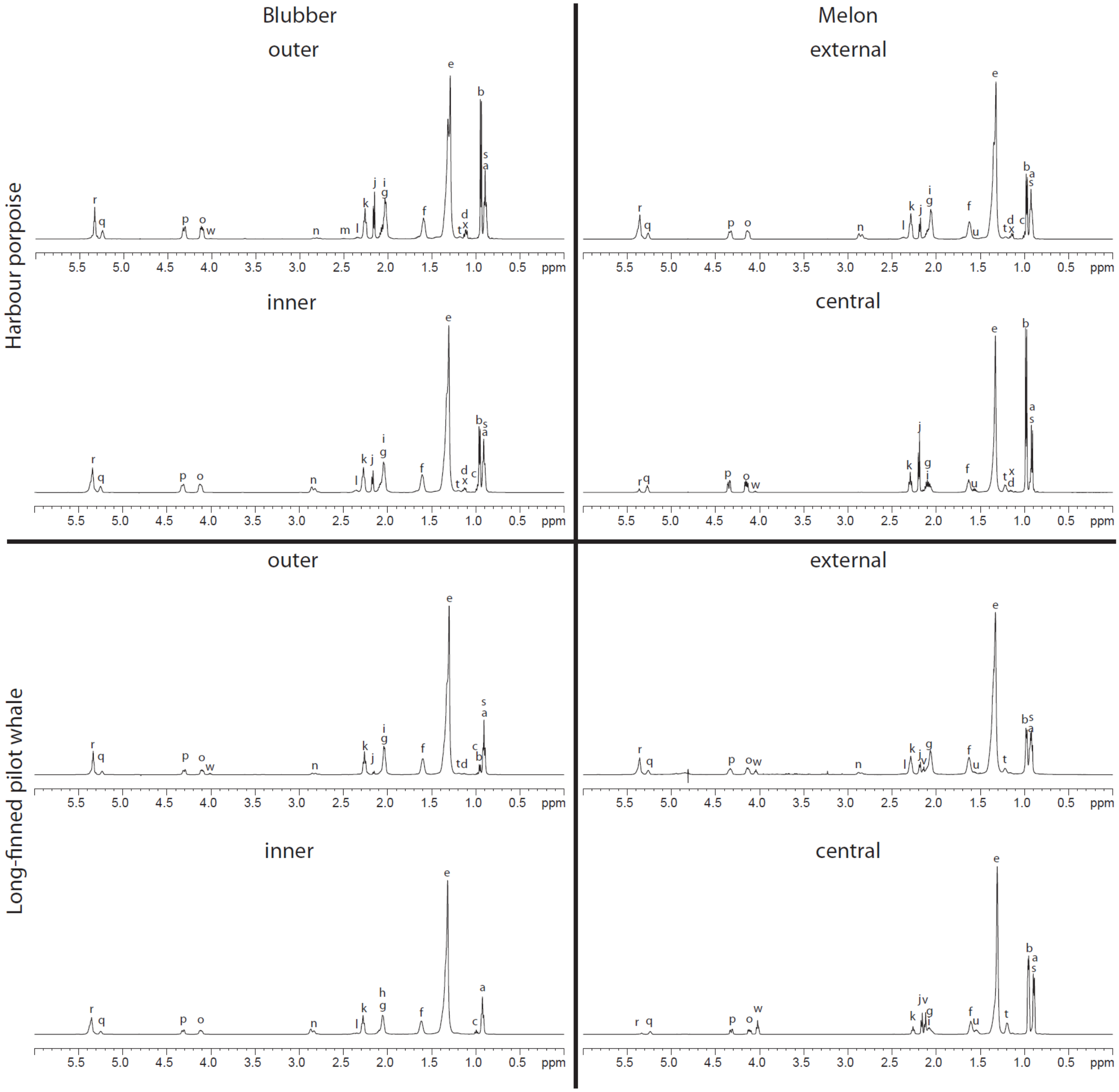

S1 Fig. ^{1}H HR-MAS spectra of the 8 samples analyzed.
Intact tissues were placed in a zirconium oxide MAS rotor, D_2O was added for ^{2}H field locking and ^{1}H HR-MAS NMR spectra were acquired at room temperature (spinning speed = 5000 Hz and ns = 64). The assignment of peaks *a* to *x* is given in Table 2.
