# Supplementary material for: Qualitative and quantitative study of the highly specialized lipid tissues of cetaceans using HR-MAS NMR and classical GC
Source: PLoS One. 2017 Jul 5;12(7):e0180597. doi: 10.1371/journal.pone.0180597 (PMC5498043; doi:10.1371/journal.pone.0180597)
Supplement: S2 Fig — Class of neutral lipids (A) and types of fatty acids (B) of 3 samples (harbour porpoise inner and outer blubbers, and pilot whale central melon) were quantitatively analyzed in 3 independent experiments. Amounts are given in % of total. IBA+2MBA, relative amount of isobutyric and 2-methylbutyric acids; IVA, relative amount of isovaleric acid; ω3 FA, relative amount of omega3-fatty acids; Iso FA, relative amount of isobranched fatty acids; Linear FA, relative amount of linear acids. (PDF) [file pone.0180597.s002.pdf]

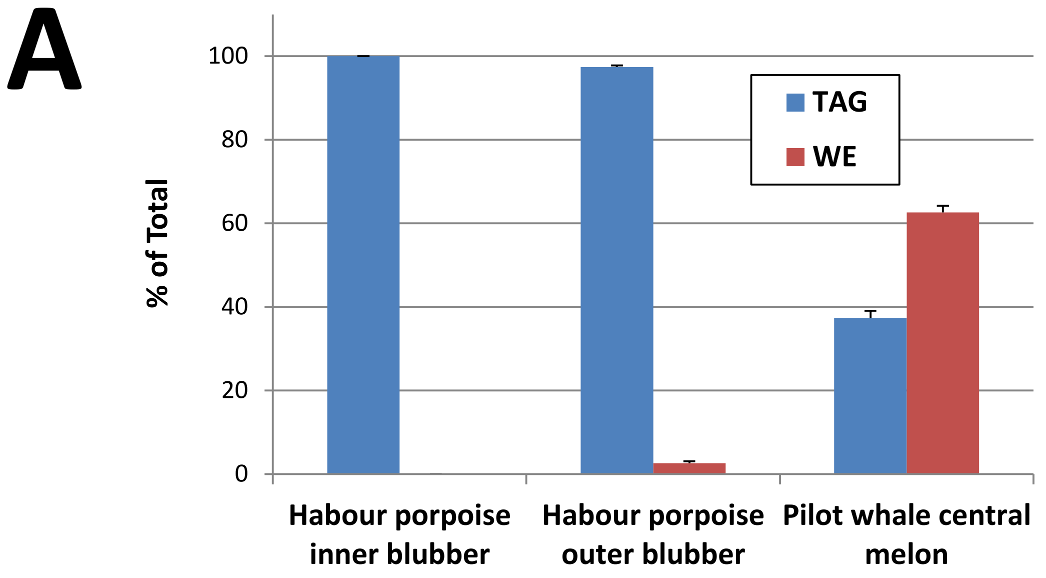

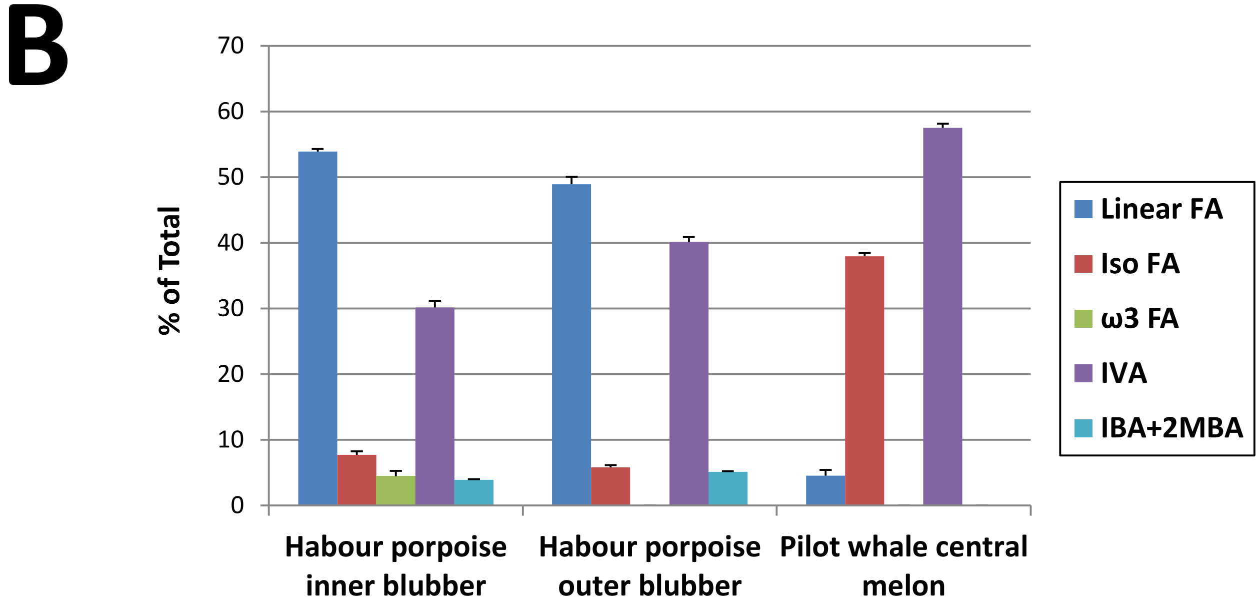

S2 Fig. Reproducibility of the HR-MAS NMR quantitative analysis.
Class of neutral lipids (A) and types of fatty acyl-chains (B) of 3 samples (harbour porpoise inner and outer blubbers, and pilot whale central melon) were quantitatively analyzed in 3 independent experiments. Amounts are given in % of total. IBA+2MBA, relative amount of isobutyric and 2-methylbutyric acids; IVA, relative amount of isovaleric acid; ω3 FA, relative amount of omega3-fatty acids; Iso FA, relative amount of isobranched fatty acids; Linear FA, relative amount of linear acids.
