# Supplementary material for: Qualitative and quantitative study of the highly specialized lipid tissues of cetaceans using HR-MAS NMR and classical GC
Source: PLoS One. 2017 Jul 5;12(7):e0180597. doi: 10.1371/journal.pone.0180597 (PMC5498043; doi:10.1371/journal.pone.0180597)
Supplement: S3 Fig — (A) IVA-isohexadecyl ester (21Carbon-Wax ester: isoC5-isoC16OH); (B) IVA-isopentadecyl ester (20Carbon-Wax ester: isoC5-isoC15OH); (C) IBA-isopentadecyl ester (19Carbon-Wax ester: isoC4-isoC15OH); (D) IBA-hexadecyl ester (20Carbon-Wax ester: isoC4-isoC16OH). (PDF) [file pone.0180597.s003.pdf]

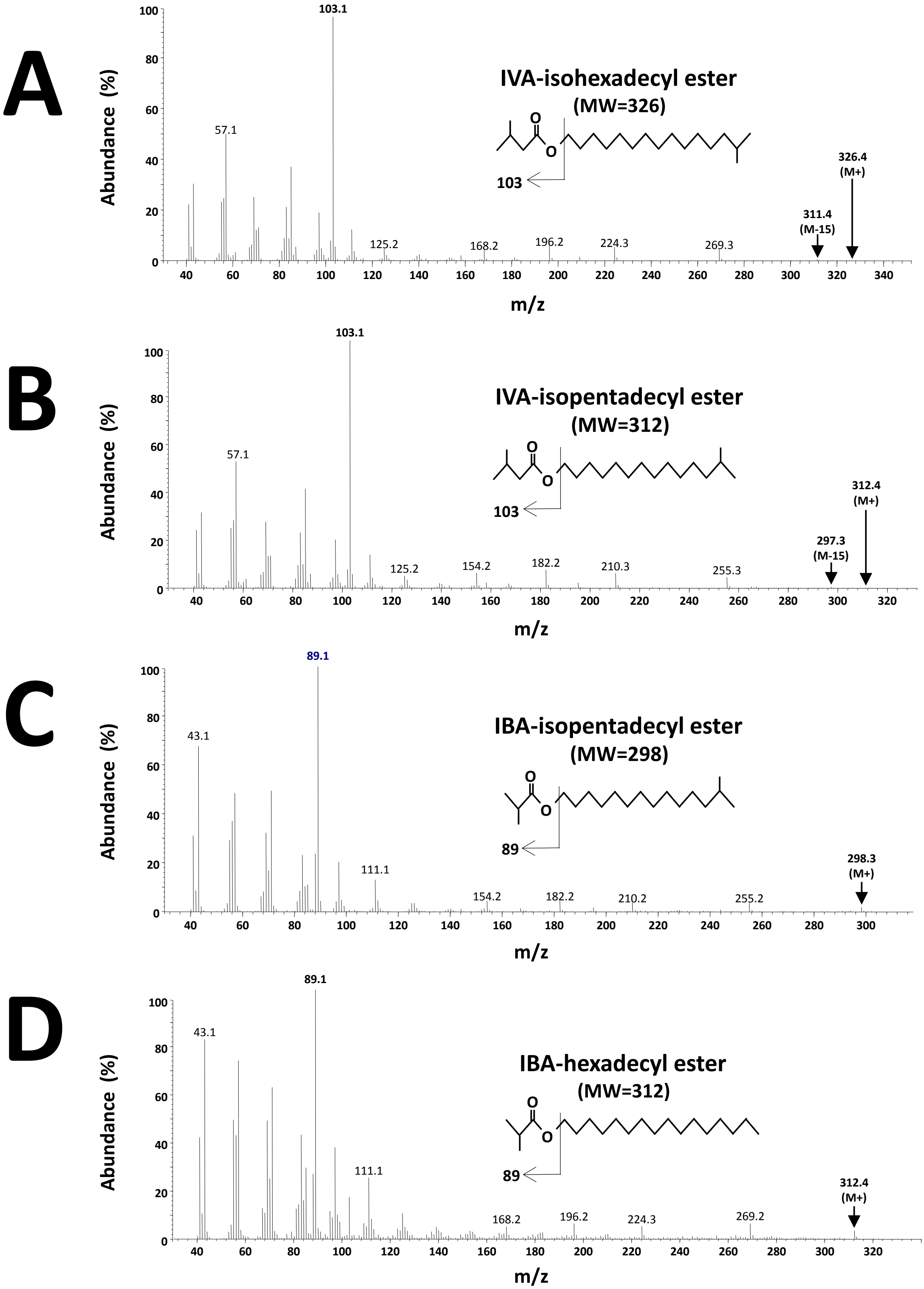

S3 Fig. Mass spectra and drawing representing fragmentation of short-chain iso fatty acids-containing wax esters.

(A) IVA-isohexadecyl ester (21Carbon-Wax ester: isoC5-isoC16OH)

(B) IVA-isopentadecyl ester (20Carbon-Wax ester: isoC5-isoC15OH)

(C) IBA-isopentadecyl ester (19Carbon-Wax ester: isoC4-isoC15OH)

(D) IBA-isohexadecyl ester (20Carbon-Wax ester: isoC4-C16OH)
