# Supplementary material for: Qualitative and quantitative study of the highly specialized lipid tissues of cetaceans using HR-MAS NMR and classical GC
Source: PLoS One. 2017 Jul 5;12(7):e0180597. doi: 10.1371/journal.pone.0180597 (PMC5498043; doi:10.1371/journal.pone.0180597)
Supplement: S1 Table — (PDF) [file pone.0180597.s004.pdf]

S1 Table. Origin of the 8 samples analyzed.

Species	Individual	Conservation state	Length	Tissue	Sample
Long-finned pilot whale *(Globicephala melas)*	Gm081211	very fresh*	5 m	blubber<br>melon	inner and outer<br>central and external
Habour porpoise *(Phocoena phocoena)*	Pp111204<br>Pp210911	very fresh*<br>very fresh*	1,75 m<br>1,6 m	blubber<br>melon	inner and outer<br>central and external

* means that carcasses were recently stranded (death of the animal within the two previous days) and did not show any visual sign of decay.
