# Supplementary material for: Qualitative and quantitative study of the highly specialized lipid tissues of cetaceans using HR-MAS NMR and classical GC
Source: PLoS One. 2017 Jul 5;12(7):e0180597. doi: 10.1371/journal.pone.0180597 (PMC5498043; doi:10.1371/journal.pone.0180597)
Supplement: S2 Table — Amounts are given in mol% and each value is the mean +/- SD from at least three replicates. n.d., not detected. (PDF) [file pone.0180597.s005.pdf]

S2 Table. Fatty acyl composition of the 8 samples according to gas chromatography analyses. Amounts are given in mol% and each value is the mean +/- SD from at least three replicates. n.d., not detected.

Mol%	Blubber				Melon			
	Habour porpoise		Pilot whale		Habour porpoise		Pilot whale	
	Outer	Inner	Outer	Inner	External	Central	External	Central
**Fatty Acids**								
12:0	1,98 +/- 0,073	1,98 +/- 0,299	1,37 +/- 0,704	0,36 +/- 0,067	n.d.	n.d.	n.d.	n.d.
12:1	1,21 +/- 0,218	0,84 +/- 0,230	0,31 +/- 0,051	0,05 +/- 0,076	n.d.	n.d.	n.d.	n.d.
13:0	0,68 +/- 0,114	1,66 +/- 1,209	n.d.	n.d.	n.d.	n.d.	n.d.	n.d.
14:0	6,41 +/- 0,842	7,37 +/- 0,012	6,94 +/- 0,123	10,23 +/- 0,282	9,74 +/- 1,0	8,19 +/- 0,7	2,07 +/- 0,4	5,01 +/- 0,4
14:1	8,28 +/- 0,153	7,72 +/- 0,035	3,12 +/- 0,043	0,72 +/- 0,186	0,50 +/- 0,1	4,69 +/- 0,1	0,16 +/- 0,1	1,88 +/- 0,1
15:0	0,56 +/- 0,039	0,62 +/- 0,033	0,76 +/- 0,088	0,86 +/- 0,122	0,61 +/- 0,0	0,64 +/- 0,0	0,27 +/- 0,0	0,56 +/- 0,1
15:1	0,33 +/- 0,016	0,32 +/- 0,008	n.d.	n.d.	n.d.	n.d.	n.d.	n.d.
16:0	4,58 +/- 0,821	5,26 +/- 0,315	14,18 +/- 0,546	19,83 +/- 0,248	6,03 +/- 1,0	6,10 +/- 0,8	2,10 +/- 0,4	7,08 +/- 0,6
16:1	26,48 +/- 0,666	20,42 +/- 0,758	24,11 +/- 0,062	12,66 +/- 0,045	5,82 +/- 0,2	23,36 +/- 0,4	1,57 +/- 0,2	15,22 +/- 0,3
17:0	0,37 +/- 0,013	0,26 +/- 0,038	0,55 +/- 0,086	0,61 +/- 0,076	0,15 +/- 0,1	0,15 +/- 0,0	0,09 +/- 0,0	0,43 +/- 0,1
17:1	0,55 +/- 0,011	0,71 +/- 0,027	0,93 +/- 0,188	0,73 +/- 0,030	0,17 +/- 0,2	0,67 +/- 0,0	0,01 +/- 0,0	0,73 +/- 0,0
18:0	0,47 +/- 0,033	0,91 +/- 0,139	1,82 +/- 0,302	3,34 +/- 0,394	0,26 +/- 0,1	0,77 +/- 0,1	0,22 +/- 0,1	1,79 +/- 0,3
18:1	13,00 +/- 1,893	20,21 +/- 0,016	35,29 +/- 0,457	36,04 +/- 0,626	0,88 +/- 1,2	19,84 +/- 1,1	0,39 +/- 0,4	21,94 +/- 1,7
18:2	0,37 +/- 0,209	1,32 +/- 0,015	0,87 +/- 0,102	0,98 +/- 0,124	0,88 +/- 1,2	0,92 +/- 0,4	0,17 +/- 0,3	0,52 +/- 0,1
**Very long-chain Fatty Acids**								
20:0	0,01 +/- 0,015	0,07 +/- 0,004	0,02 +/- 0,032	0,22 +/- 0,019	n.d.	0,03 +/- 0,0	n.d.	0,15 +/- 0,0
20:1	0,70 +/- 0,131	3,51 +/- 0,614	1,57 +/- 0,127	4,48 +/- 0,762	n.d.	2,55 +/- 0,4	n.d.	1,46 +/- 0,4
20:2	n.d.	0,29 +/- 0,017	0,09 +/- 0,123	0,33 +/- 0,054	n.d.	0,22 +/- 0,3	n.d.	0,02 +/- 0,0
20:4ω3	0,07 +/- 0,081	0,86 +/- 0,093	0,15 +/- 0,043	0,06 +/- 0,091	n.d.	0,80 +/- 0,9	n.d.	0,17 +/- 0,0
20:5ω3 (EPA)	0,12 +/- 0,064	0,21 +/- 0,058	0,38 +/- 0,017	0,43 +/- 0,015	n.d.	0,34 +/- 0,3	n.d.	0,47 +/- 0,1
22:0	0,00 +/- 0,000	0,00 +/- 0,000	0,03 +/- 0,022	0,09 +/- 0,066	n.d.	n.d.	n.d.	0,23 +/- 0,0
22:1	0,21 +/- 0,032	1,77 +/- 0,271	0,36 +/- 0,013	2,62 +/- 0,385	n.d.	1,21 +/- 0,0	n.d.	0,39 +/- 0,0
22:5ω3	n.d.	0,48 +/- 0,094	0,14 +/- 0,091	0,67 +/- 0,274	n.d.	0,17 +/- 0,2	n.d.	0,21 +/- 0,0
22:6ω3 (DHA)	n.d.	2,06 +/- 0,510	0,25 +/- 0,186	0,96 +/- 0,297	n.d.	0,52 +/- 0,7	n.d.	0,46 +/- 0,1
24:1	n.d.	0,14 +/- 0,037	0,05 +/- 0,008	0,28 +/- 0,116	n.d.	n.d.	n.d.	n.d.
**Branched Fatty Acids**								
5:0 iso (IVA)	29,17 +/- 1,773	16,09 +/- 1,336	3,24 +/- 1,648	0,47 +/- 0,258	45,32 +/- 3,7	22,40 +/- 1,1	42,22 +/- 2,7	19,15 +/- 3,1
13:0 iso	n.d.	n.d.	n.d.	n.d.	2,13 +/- 0,2	0,51 +/- 0,0	0,55 +/- 0,1	2,14 +/- 2,6
14:0 iso	0,76 +/- 0,237	0,54 +/- 0,139	n.d.	n.d.	3,04 +/- 0,3	0,80 +/- 0,1	2,44 +/- 0,3	1,24 +/- 0,1
15:0 iso	1,06 +/- 0,112	1,12 +/- 0,119	n.d.	n.d.	17,45 +/- 0,6	2,16 +/- 0,4	12,91 +/- 0,9	5,87 +/- 0,4
15:0 anteiso	0,41 +/- 0,032	0,52 +/- 0,034	n.d.	n.d.	n.d.	n.d.	n.d.	n.d.
16:0 iso	0,48 +/- 0,027	0,25 +/- 0,105	n.d.	n.d.	2,61 +/- 0,3	0,85 +/- 0,1	2,00 +/- 0,2	1,19 +/- 0,3
17:0 iso	n.d.	n.d.	n.d.	n.d.	0,54 +/- 0,1	0,31 +/- 0,0	0,24 +/- 0,0	0,11 +/- 0,2
**Fatty Alcohols**								
14:0-OH	n.d.	n.d.	n.d.	n.d.	0,01 +/- 0,0	n.d.	0,82 +/- 0,0	0,25 +/- 0,2
15:0-OH	n.d.	n.d.	n.d.	n.d.	0,02 +/- 0,0	n.d.	0,99 +/- 0,1	0,29 +/- 0,3
16:0-OH	n.d.	n.d.	0,48 +/- 0,075	0,10 +/- 0,148	n.d.	n.d.	n.d.	n.d.
16:1-OH	n.d.	n.d.	n.d.	n.d.	0,75 +/- 0,0	0,09 +/- 0,1	3,74 +/- 0,4	1,13 +/- 1,0
18:0-OH	0,34 +/- 0,068	0,04 +/- 0,055	0,26 +/- 0,115	0,29 +/- 0,116	0,10 +/- 0,1	0,49 +/- 0,3	0,12 +/- 0,0	n.d.
18:1-OH	0,15 +/- 0,081	0,42 +/- 0,357	0,82 +/- 0,767	0,11 +/- 0,150	0,18 +/- 0,1	0,28 +/- 0,1	0,46 +/- 0,1	0,51 +/- 0,5
20:1-OH	n.d.	n.d.	n.d.	n.d.	n.d.	n.d.	n.d.	n.d.
**Branched Fatty Alcohols**								
15:0-OH iso	n.d.	n.d.	n.d.	n.d.	0,20 +/- 0,3	n.d.	13,81 +/- 0,9	4,36 +/- 2,0
16:0-OH iso	n.d.	n.d.	n.d.	n.d.	0,44 +/- 0,1	n.d.	10,83 +/- 0,3	3,83 +/- 0,9
17:0-OH iso	n.d.	n.d.	n.d.	n.d.	0,04 +/- 0,1	n.d.	1,18 +/- 0,1	0,24 +/- 0,2
**Others**								
Unidentified	1,26 +/- 0,557	2,04 +/- 0,229	1,91 +/- 0,218	2,47 +/- 0,431	2,14 +/- 0,8	0,93 +/- 0,2	0,63 +/- 0,1	1,00 +/- 1,0
